# Regulatory role of IL-17-mediated inflammatory pathways in lung cancer initiation and progression

**DOI:** 10.1007/s12672-026-04884-7

**Published:** 2026-03-26

**Authors:** Xinjia Xu, Xinan Lu, Wei Wu, Xinyi Chen, Aijun Zhang

**Affiliations:** Respiratory and Critical Care Medicine, Cixi People’s Hospital, Wenzhou Medical University, Ningbo, China

**Keywords:** Interleukin-17, Lung cancer, Targeted therapy, Inflammation, Cytokines, Immune checkpoint inhibitors

## Abstract

Lung cancer continues to present a substantial global health and economic challenge, with consistently high rates of incidence and mortality. While there have been significant strides in precision diagnostics and treatment methods that have contributed to a reduction in mortality over previous years, progress has plateaued recently, largely due to complications such as side effects and resistance to therapies. Emerging research indicates that interleukin-17 (IL-17), a cytokine with pro-inflammatory properties, plays a critical role in various stages of lung cancer, including initiation, growth, invasion, metastasis, angiogenesis, lymphangiogenesis, and treatment resistance. This positions IL-17 as both a crucial regulator and a valuable prognostic marker for the disease’s progression. This review aims to provide an overview of the most recent developments in IL-17 research as it relates to lung cancer, offering a theoretical framework for its potential as a therapeutic target in both the prevention and treatment of lung cancer.

## Introduction

 Lung cancer is one of the most prevalent types of malignancies, characterized by high rates of both incidence and mortality. The “2022 Global Cancer Statistics” report reveals that in 2022, 2.48 million new lung cancer cases were diagnosed worldwide, with nearly 1.81 million deaths attributed to the disease, representing 18.7% of all cancer-related fatalities [1–3]. This positions lung cancer as the leading cause of cancer-related mortality globally. However, in recent years, the introduction of precision medicine and advancements in immunotherapy have contributed significantly to a reduction in the mortality rate for lung cancer [4–6].

Precision medicine, through genetic testing and matched targeted therapies against driver mutations like EGFR and ALK, enables personalized treatment strategies that significantly improve therapeutic outcomes [7–10]. In parallel, immune checkpoint inhibitors like PD-1/PD-L1 inhibitors have notably strengthened the immune system’s ability to attack tumors, thereby improving the prognosis of patients suffering from advanced lung cancer [11–13]. Furthermore, improvements in early screening techniques and minimally invasive surgical procedures have played a pivotal role in the early detection and treatment of lung cancer, resulting in a further decrease in its mortality rate [14–17].

Immunotherapy, while effective, is also frequently linked with immune-related side effects such as immune pneumonitis and hepatitis, which may negatively impact the patient’s quality of life [18–20]. As a result, identifying reliable biomarkers and therapeutic targets is critical for lung cancer diagnosis and treatment, forming the foundation for the development of targeted and immuno-precision therapies.

Specifically, EGFR (epidermal growth factor receptor) mutations, especially in non-small cell lung cancer (NSCLC), are closely associated with tumor growth and resistance to treatment. KRAS mutations, commonly seen in smoking-related lung adenocarcinoma, contribute to tumor proliferation and metastasis. Additionally, rearrangements of the ALK (anaplastic lymphoma kinase) gene have been detected in a small subset of lung cancers in non-smokers, and these cancers are sensitive to targeted therapies. Furthermore, alterations in the p53 gene and disruptions in the PI3K/AKT/mTOR signaling pathway are strongly linked to the onset and progression of lung cancer, promoting cell survival and proliferation, and may also contribute to the development of resistance to treatment [21–23].

**Fig. 1:**
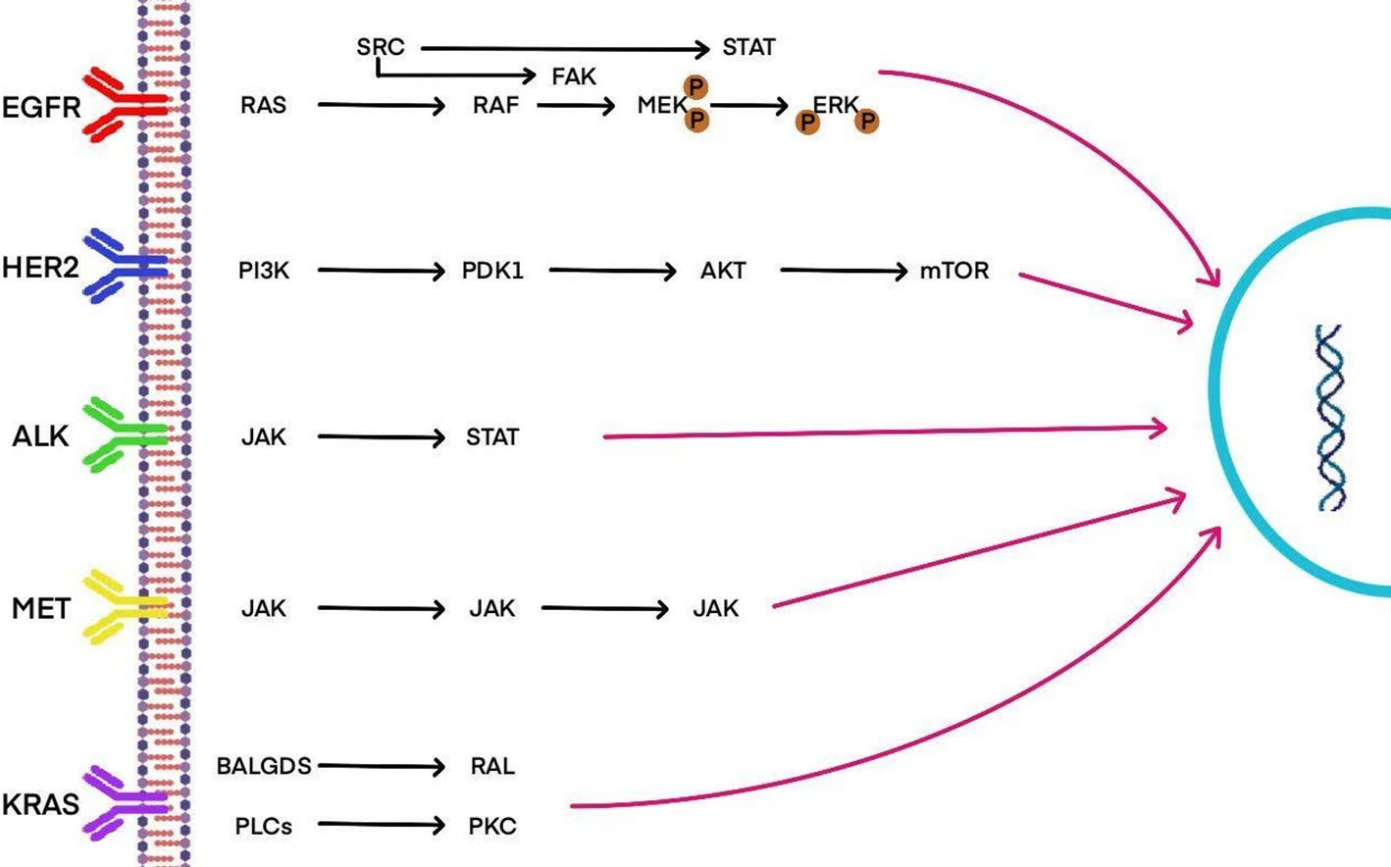
Molecular pathways involved in the development and progression of lung cancer

In summary, mutations in these genes and signaling pathways represent crucial biomarkers and therapeutic targets, facilitating early diagnosis and the targeted treatment of lung cancer (Fig. 1). In this context, IL-17 emerges not only as an inflammatory cytokine but also as a central coordinator of disease pathogenesis, acting through three interconnected axes: (1) as a potent driver of chronic inflammation that promotes tumorigenesis, (2) As a key regulator of tumor microenvironment (TME), it affects the function of immune cells, angiogenesis and metastasis, And (3) as a key mediator of resistance to multiple therapies, including chemotherapy, targeted therapy, and immunotherapy, and a contributor to treatment-related toxicity. This IL-17-centric regulatory network integrates inflammatory signaling and core oncogenic processes, positioning it as a key node for prognostic assessment and therapeutic intervention.

Interleukin-17 (IL-17) is a pro-inflammatory cytokine, mainly released by Th17 cells, playing a pivotal role in immune responses. It promotes inflammation, facilitates immune cell recruitment, and contributes to tissue repair. IL-17 is not only vital in numerous immune-related diseases but also plays a critical role within the tumor’s immune microenvironment [24–26]. For example, IL-17-generated pseudotumor cells, T cells, and neutrophils synergistically promote breast cancer metastasis [27], IL-17 A promotes tumorigenesis and upregulates PD-L1 expression in non-small cell lung cancer [28], Blocking IL-17 A enhances the response of colorectal cancer to PD-1-based immunotherapy [29]. In lung cancer, IL-17 has been implicated in immune evasion by tumors and promotes tumor cell proliferation, invasion, metastasis, angiogenesis, and lymphangiogenesis [30]. The role of IL-17 in immune regulation is closely related to the regulation of FOXO transcription factor. FOXO protein is a key regulator of cellular stress response, apoptosis, metabolism and immune homeostasis. Frontier studies have shown a bidirectional regulatory relationship between IL-17 and FOXO: IL-17 regulates inflammatory gene expression and cell survival in part by inactivating FOXO1/3a, while FOXOs in turn inhibit Th17 differentiation and IL-17 production [26]. Therefore, the IL-17/FOXO axis constitutes a core node connecting chronic inflammation, cell fate determination and immune dysregulation, and its potential role in the occurrence, progression and treatment resistance of lung cancer deserves further exploration. Inhibition of pathways such as STAT3, NF-κB, and AP-1 by Notopterol suppresses IL-17-induced proliferation and invasion in A549 lung adenocarcinoma cells [31], IL-17 also promotes non-small cell lung cancer metastasis by enhancing MMP9 gene transcription and expression through GCN5-dependent SOX4 acetylation [32]. Furthermore, IL-17 may influence how lung cancer responds to treatment, with certain studies linking it to drug resistance. As a result, IL-17 serves a dual function in the initiation, progression, and prognosis of lung cancer, acting both as an essential regulator of tumor growth and as a potential therapeutic target [33]. It may also help reduce the side effects of immunotherapy. Therefore, IL-17 holds promise as a crucial molecule in future strategies for lung cancer treatment.

### IL-17 families

**Fig. 2:**
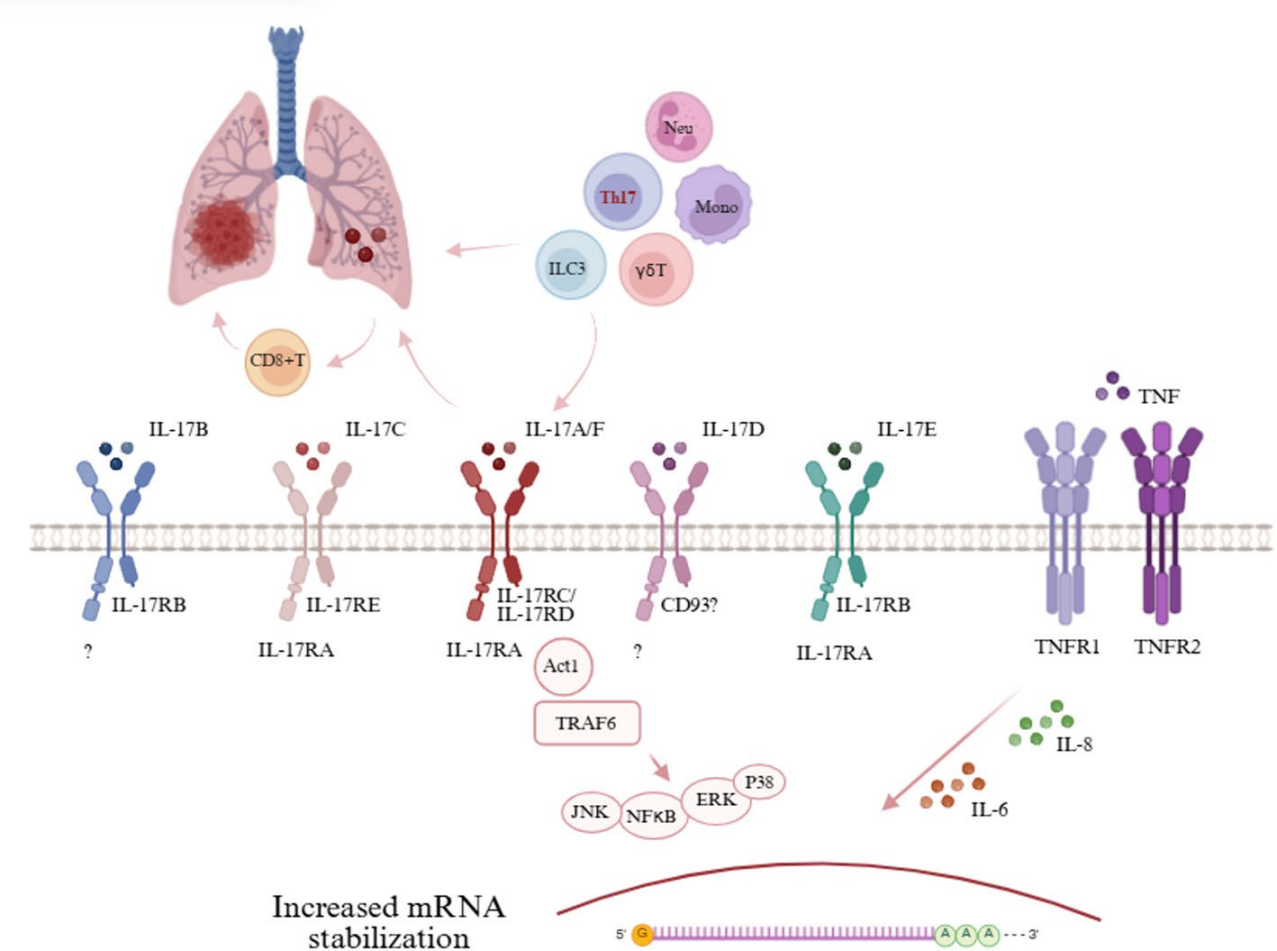
The overview of IL-17

IL-17 was first identified by researchers in 1993, initially designated as cytotoxic T lymphocyte antigen 8 (CTLA-8) [34]. Subsequently, it was discovered that Th17 cells, a crucial subset of CD4 + T cells, secreted CTLA-8. Following the renumbering of interleukins, this cytokine was renamed IL-17 [35, 36]. Over time, additional cytokines sharing homology with IL-17 were discovered. To distinguish them, the first identified IL-17 was referred to as IL-17 A. Presently, the IL-17 family consists of six distinct members: IL-17 A, IL-17B, IL-17 C, IL-17D, IL-17E (also known as IL-25), and IL-17 F. Among these, IL-17 A and IL-17 F exhibit the greatest sequence homology, approximately 50%, which results in overlapping functional roles [37–39]. The IL-17 receptor (IL-17R) family comprises five receptor subunits: IL-17RA, IL-17RB, IL-17RC, IL-17RD, and IL-17RE. These subunits share structural similarities, including an extracellular fibronectin type III-like domain, a conserved cytoplasmic SEF/IL-17R domain, and a distal activation domain. Upon assembly, the IL-17 receptor subunits form various heterodimers that bind IL-17 ligands, triggering downstream signaling pathways and initiating a cascade effect. Among these, the heterodimer composed of IL-17RA and IL-17RC functions as the primary co-receptor for IL-17 A and IL-17 F, playing a pivotal role in the progression of disease **(**Fig. 2**)** [40–42]. IL-17 A and IL-17 F can each form homodimers or co-assemble into a heterodimeric IL-17 A–IL-17 F complex. These dimeric ligands bind to the IL-17 receptor (IL-17R) complex, which is composed of the IL-17RA and IL-17RC chains. Upon receptor activation, signaling pathways are triggered, involving the adaptor protein ACT1, nuclear factor-kappa B (NF-κB), and tumor necrosis factor (TNF) receptor-associated factor 6 (TRAF6), ultimately leading to the enhanced transcription of IL-6 and IL-8 genes. TNF, existing as a homotrimer, interacts with TNF receptors 1 (TNFR1) and 2 (TNFR2). The combination of IL-17 ligands and TNF often results in a synergistic effect, which can be partly attributed to increased mRNA stability and the overexpression of TNFR.

Research has demonstrated that, Th17 cells are the primary source of IL-17, but other cells such as neutrophils, monocytes, group 3 innate lymphoid cell (ILC3) and γδ T cells can also secrete small amounts of IL-17 [43–45]. The diverse sources and targets of IL-17 contribute to its “double-edged sword” function in tumor regulation. On one side, IL-17 enhances tumor growth by increasing the expression of cytokines like vascular endothelial growth factor (VEGF) and transforming growth factor-beta (TGF-β). On the other side, IL-17 activates immune cells, thereby promoting tumor cell destruction. IL-17 exhibits different effects across various types of tumors. In lung, liver, and breast cancer, IL-17 primarily facilitates tumor progression [46–48], while in esophageal cancer, it exerts a suppressive effect [49]. Importantly, the functional duality of IL-17 in the tumor microenvironment depends not only on the context but also on its cellular origin. IL-17 derived from Th17 cells and neutrophils generally promotes inflammation, angiogenesis, and immunosuppression, thereby contributing to tumor progression [50]. In contrast, IL-17 produced by innate lymphoid cells such as γδ T17 cells or ILC3s can exhibit anti-inflammatory, tissue repair and even anti-tumor properties [51]. This distinction implies that the goal of therapeutic strategies should be not only to block IL-17 alone, but also to selectively target the pathogenic source and block the pro-inflammatory pro-tumor developing IL-17 (Table 1).

**Table 1:** The IL-17 family and its receptors

IL-17 Family Members	Receptor	Pharmacological Effects	Clinical/Preclinical Applications	Reference
IL-17 A	IL-17RA–IL-17RC or IL-17RA–IL-17RD	Regulates inflammatory responses, induces cytokine release, modulates autoimmune diseases and chronic inflammation	Treatment of psoriasis, rheumatoid arthritis, ankylosing spondylitis and other autoimmune diseases	[52]
IL-17B	IL-17RB	Regulates inflammatory responses, anti-tumor effects	–	[53, 54],
IL-17 C	IL-17RE–IL-17RA	Regulates mucosal immunity, promotes Th17 cell activation and inflammatory responses, regulates intestinal and lung immune defense	Enteritis, colorectal cancer and chronic obstructive pulmonary disease	[55, 56]
IL-17D	CD93	May participate in regulating inflammation and immune response, liver injury repair	–	[52]
IL-17E (IL-25)	IL-17RA–IL-17RB	Activates type 2 immune responses, promotes cytokine secretion, involved in allergic asthma, eosinophilic granulomatous inflammation	Asthma, atopic dermatitis and other type 2 inflammatory diseases treatment	[57–59]
IL-17 F	IL-17RA–IL-17RC	Induces inflammatory cytokine expression, involved in psoriasis, inflammatory bowel disease, etc.	Treatment of psoriasis and Crohn’s disease	[52]

### Mechanisms by which IL-17 regulates lung cancer progression

In 2013, a study demonstrated that single nucleotide polymorphisms (SNPs) within IL-17 A and IL-17 F contribute to heightened lung cancer susceptibility. Notably, the 7488AG variant of IL-17 F was correlated with both a greater incidence of lung malignancy and an increased tendency for advanced or metastatic presentation [60]. Similarly, research by Yanchao He and colleagues identified a significant positive relationship between the rs2275913 polymorphism in IL-17 A and lung cancer risk. Further subgroup analyses revealed that smokers harboring homozygous mutations at rs2275913 (IL-17 A) and rs12203582 (IL-17 F) exhibited a higher propensity for developing lung cancer [61].

Meta-analyses, which integrate data across independent investigations through statistical synthesis, have become an essential approach for assessing disease predisposition and therapeutic effectiveness. Recent clinical meta-analysis findings highlighted four SNPs—IL-17 A rs2275913, IL-17 A rs8193037, IL-17 F rs1889570, and IL-17 F rs763780—as being linked to lung cancer risk. Additionally, weak linkage disequilibrium (LD) between IL-17 A rs2275913 and rs8193037 was detected in East Asian cohorts, whereas no such association emerged in European or African populations [62]. These results imply a possible ethnicity-specific role of IL-17 in lung carcinogenesis.

Among histopathological subtypes of non-small cell lung cancer (NSCLC), lung adenocarcinoma (LUAD) predominates, with epidermal growth factor receptor (EGFR) alterations representing the most frequent driver mutations. An investigation in Taiwanese LUAD patients revealed that the rs8193037 variant of IL-17 A was significantly associated with a reduced likelihood of EGFR mutations in smokers (*P* = 0.035). Conversely, within the wild-type EGFR subgroup, carriers of the rs8193036 polymorphism in IL-17 A were more likely to present with advanced disease stage (*P* = 0.016) and lymphatic spread (*P* = 0.049) [63].

Collectively, these findings strongly support the association of IL-17 A rs2275913 and rs8193037 polymorphisms with lung cancer risk, while evidence concerning other loci remains inconclusive. A large-scale study in 2019 examining IL-17 gene family alterations and clinical outcomes in 5,238 lung cancer cases reported higher frequencies of IL-17 A, IL-25, and IL-17 F mutations (2.0%, 2.1%, and 1.9%, respectively), predominantly due to gene amplification events. In comparison, mutations in IL-17B, IL-17 C, and IL-17D were less common (0.8%, 1.1%, and 1.1%, respectively). Interestingly, IL-17D, IL-25, and IL-17 F alterations were more prevalent in lung squamous cell carcinoma (LUSC), whereas LUAD cases more frequently harbored mutations in IL-17 A, IL-17B, and IL-17 C. This pattern suggests subtype-specific functional roles for IL-17 [64].

In summary, substantial epidemiological and genetic evidence indicates that single nucleotide polymorphisms (SNPs) in the IL-17 gene family, particularly IL-17 A and IL-17 F, are closely associated with lung cancer susceptibility, with this association exhibiting significant heterogeneity across ethnicities, smoking status, and histological subtypes. These genetic variations not only influence individual predisposition to lung cancer but may also contribute to determining the tumor’s driver mutation profile (e.g., EGFR status) and clinical progression features such as disease stage and lymphatic spread. Furthermore, the differential mutation frequencies of IL-17 family members across non-small cell lung cancer (NSCLC) subtypes suggest subtype-specific functional roles in lung tumorigenesis. These findings provide crucial insights into the role of inflammatory cytokines in the early stages of lung cancer development. However, genetic predisposition represents only one aspect of IL-17’s involvement in lung cancer, deeper mechanistic insights lie in the direct regulation of various cellular behaviors within the tumor microenvironment by IL-17. Therefore, the following section will focus on how IL-17 regulates key malignant phenotypes of lung cancer cells—specifically proliferation, invasion, and migration—systematically elucidating its direct biological functions in tumor progression and thereby revealing the complex regulatory network through which IL-17 influences lung cancer development.

### Regulation of lung cancer cell proliferation by IL-17

Extensive research has established a link between IL-17 and both the development of lung cancer and its unfavorable prognosis. Nonetheless, its precise influence on the proliferation of lung cancer cells remains debated. Multiple investigations have indicated that stimulation with IL-17 A does not markedly influence lung cancer cell growth. One study, for instance, showed that altering IL-17RA expression—either by silencing or overexpressing it—did not modify the cells’ proliferation rate [65]. Moreover, since IL-17 is naturally synthesized and can also be externally administered. An in vitro study has shown that exogenous IL-17 A or IL-17 F treatment does not impair the survival ability of lung cancer cells such as A549 and LLC1 [66].

Contrary to these observations, later studies presented opposing evidence. IL-17 A exposure was shown to markedly elevate HMGA1 levels, which in turn interacted with the Cyclin D1 promoter to stimulate its transcription, thereby enhancing cell proliferation [67]. Additional research revealed that exogenous IL-17 also facilitated lung cancer cell growth and motility by activating the NF-κB signaling cascade. Similar results were reported by Lee K’s research group, who explored how IL-17 signaling contributes to EGFR-TKI therapy. Findings by Lee K’s team showed that, in EGFR-mutant NSCLC cells, the IL-17 A/IL-17RC signaling axis enhanced phosphorylation of both EGFR and Met, promoting proliferation and resistance [68]. Lastly, Notopterol—a furanocoumarin with strong antitumor efficacy—has been reported to suppress lung cancer cell proliferation by decreasing IL-17 levels and reducing the expression of cell cycle regulators in vitro, including Cyclin D1 [31]. Nonetheless, as these studies did not examine the effects of endogenously overexpressed IL-17, the robustness of these findings remains questionable, making it difficult to definitively resolve the conflicting results.

In summary, the regulatory role of IL-17 in lung cancer cell proliferation exhibits highly complex and seemingly contradictory characteristics, with existing research findings showing significant divergence. In summary, the apparent paradox of IL-17 actions may stem from key differences between studies: acute exogenous stimuli in vitro versus chronic endogenous signaling in vivo, tumor-intrinsic effects versus microenvironment-mediated mechanisms, and simplified in vitro cell models versus complex samples from real-world patient sources (Table 2).

Although IL-17 is not a canonical immune checkpoint molecule, it exerts a highly context-dependent dual role in tumor immunoregulation. Recent studies have begun to uncover the mechanistic basis for this functional plasticity. For instance, a study demonstrated that patients who developed immune-related adverse events (irAEs)—such as rash, colitis, or myocarditis—following immune checkpoint inhibitor (ICI) therapy exhibited significant enrichment of IL-17 A⁺ CD4⁺ T cells (Th17 cells) in both peripheral blood and affected tissues. Strikingly, administration of anti–IL-17 A antibodies (e.g., secukinumab) effectively ameliorated these irAEs without compromising antitumor efficacy, positioning IL-17 A as a key driver of ICI-induced toxicity and a promising target for improving therapeutic safety [69]. Conversely, IL-17 is not solely pro-inflammatory. A study evealed that in aged melanoma-bearing mice, the antitumor efficacy of anti–PD-L2 therapy critically depended on an IL-17/IFNγ axis: IL-17 secreted by CD4⁺ T cells promoted the infiltration and effector function of IFNγ⁺ CD8⁺ T cells within tumors. Notably, exogenous IL-17 supplementation was sufficient to restore responsiveness to αPD-L2 in young mice that were otherwise non-responders [70].

Together, these seemingly contradictory findings converge on a central concept: the biological outcome of IL-17 signaling is not intrinsic but is dynamically shaped by its cellular source, host age, co-expressed cytokine networks, and local microenvironmental cues. We hypothesize that IL-17 persistently secreted by stromal or myeloid cells within the tumor microenvironment (TME) preferentially activates pro-inflammatory and pro-survival pathways such as NF-κB and STAT3, thereby promoting tumor progression, in contrast, transient IL-17 release from adaptive immune cells (e.g., Th17 cells) under specific immunostimulatory conditions—such as in aged hosts or during combination immunotherapy—may enhance CD8⁺ T cell recruitment and function, exerting indirect antitumor effects. This framework offers a more nuanced and testable explanation for the inconsistent proliferative outcomes observed across different experimental models and underscores the necessity of precisely defining the spatiotemporal origin and signaling context of IL-17 in future studies.

This inconsistency may stem from multiple factors: First, differences in experimental models are crucial, including the genetic background of the lung cancer cell lines used, expression levels of IL-17 receptors (IL-17RA/RC), and the presence or absence of other cytokines in the tumor microenvironment. Second, IL-17’s effects may not directly drive proliferation but rather indirectly promote tumor growth by reshaping the microenvironment. For instance, IL-17 can induce stromal or immune cells to release growth factors and proangiogenic factors, thereby creating favorable growth conditions for tumor cells. Furthermore, IL-17 effects may exhibit dose- and time-dependent properties or require synergistic interaction with other signaling pathways to manifest its pro-proliferative function. Notably, most studies rely on exogenous IL-17 stimulation, failing to fully mimic the physiological context of endogenous, persistent inflammation—a key factor potentially contributing to inconsistent findings. Future studies urgently require more physiologically relevant models, such as co-culture systems, genetically engineered mouse models, or single-cell multi-omics analyses, to elucidate the precise functions of IL-17 within specific cell subpopulations and microenvironmental contexts. Concurrently, deeper exploration of the interactions between IL-17 signaling and other oncogenic pathways is warranted, particularly regarding their significance in the context of targeted therapy resistance. Thus, while evidence for IL-17’s direct proliferative effects remains controversial, its role as a key inflammatory hub in the tumor microenvironment—multidimensionally regulating and promoting lung cancer progression—is increasingly well-established. Consequently, there is an urgent need to integrate cutting-edge methodologies such as multi-omics, organoids, and neutralizing antibodies to comprehensively and rigorously evaluate IL-17’s specific regulatory mechanisms on lung cancer cells and other tumor cells.

**Table 2:** Summary of experimental models, IL-17 sources, and observed outcomes related to IL-17’s function in lung cancer

Experimental Model	IL-17 Source / Stimulation	Observed Outcome(s)	Reference
In Vitro (A549, LLC1 cells)	Exogenous IL-17 A or IL-17 F	No impairment of cell survival/proliferation	[64]
In Vitro (Lung cancer cells)	Altered IL-17RA expression (silencing/overexpression)	No change in proliferation rate	[63]
In Vitro	Exogenous IL-17 A	Elevated HMGA1, enhanced Cyclin D1 transcription, increased proliferation	[65]
In Vitro (EGFR-mutant NSCLC cells)	IL-17 A/IL-17RC signaling axis	Enhanced EGFR/Met phosphorylation, promoted proliferation & resistance	[66]
In Vitro (A549 cells)	Notopterol treatment (decreases IL-17)	Suppressed proliferation, reduced Cyclin D1	[31]
In Vitro (Lung cancer cells)	IL-17	Activated STAT3, increased MMP9 & MMP19, enhanced invasion	[73, 74]
In Vitro	IL-17	Upregulated GCN5, led to SOX4 acetylation, increased MMP9 transcription, facilitated migration/invasion	[32]
In Vitro	IL-17 A (activating NF-κB)	Upregulated ZEB1, boosted Vimentin, repressed E-cadherin, facilitated EMT	[78]
In Vivo (Murine NSCLC model)	IL-17	Facilitated angiogenesis and tumor growth via CXCR2-dependent mechanisms	[88]
Patient Samples (Serum, Tissue, Pleural Effusion)	Endogenous (Patient-derived)	High IL-17 expression correlated with poorer overall survival and shorter disease-free survival	[98]
In Vivo (LLC mouse model)	Endostatin treatment (reduces IL-17)	Significant reduction in IL-17 expression in tumor tissues	[96]
In Vivo (Tumor mouse model)	IL-17 blockade + checkpoint blockade	Reshaped TME, significantly suppressed tumor progression	[106]
In Vivo (LSL-K-rasG12D mouse model)	Intratracheal IL-10 (disrupts macrophage-Th17 axis)	Inhibited IL-17 + CD4+ T cell-driven tumor growth	[119]
In Vivo (Abt mouse model with LLC)	Symbiotic bacteria (mediating IL-17 response)	Impaired γδT17 response in Abt mice, restoration with normal γδT17 cells or IL-17 supplementation	[121]

### IL-17-mediated modulation of lung cancer cell invasion and migration

Metastasis constitutes a pivotal stage in the progression of lung cancer, accounting for more than 70% of lung cancer-related mortality and representing the primary reason for treatment failure [71–73]. This phenomenon is orchestrated by a variety of mechanisms, including dynamic interactions within the tumor microenvironment, the induction of epithelial-mesenchymal transition (EMT), and increased angiogenic and lymphangiogenic activity. Matrix metalloproteinases (MMPs), a group of proteolytic enzymes, collaborate with tissue inhibitors of metalloproteinases (TIMPs) under normal conditions to remodel the extracellular matrix (ECM) and maintain tissue integrity. However, abnormal expression of MMPs accelerates ECM degradation, facilitating tumor cells in breaching adjacent tissues and spreading to distant sites [74–76].

Emerging evidence indicates that IL-17 influences lung cancer cell migration by modulating MMPs through diverse signaling cascades. For instance, IL-17 has been demonstrated to activate STAT3 signaling in lung cancer cells, which in turn increases the expression of MMP9 [77] and MMP19 [78], thereby enhancing the invasive capacity of tumor cells. Additionally, the p38 MAPK pathway, a critical regulator of tumor progression, is implicated in this process: IL-17 A/IL-17RA signaling augments p38 phosphorylation in NSCLC cells, elevating MMP2 and MMP9 levels and promoting cell invasion.

Moreover, general control nonderepressible 5 (GCN5), a transcriptional coactivator with acetyltransferase functionality, and SRY-related HMG-box 4 (SOX4), a transcription factor, synergistically enhance the transcription of metastasis-related genes. IL-17 upregulates GCN5, leading to SOX4 acetylation, and these two factors jointly bind to the MMP9 promoter region (-915 to -712 nt), further increasing MMP9 transcription and facilitating lung cancer cell migration and invasion.

Epithelial-mesenchymal transition (EMT) constitutes a key biological process that enhances tumor cell invasion and metastasis. During this transition, epithelial markers, including E-cadherin, are suppressed alongside a loss of cell polarity, whereas mesenchymal markers such as N-cadherin and Vimentin are elevated, leading to diminished cell adhesion and increased motility [79–81]. An in vitro study shows, activation of NF-κB signaling by IL-17 A significantly upregulates the transcription factor ZEB1, which subsequently boosts Vimentin levels and represses E-cadherin expression, thereby facilitating EMT in lung cancer cells [82]. Moreover, NF-κB signaling elevates the expression of the downstream NLRP3 inflammasome and its associated proteins, Caspase-1 and IL-1β, which further promote EMT [83]. The above results indicate that NLRP3 participates in the migration, invasion and the EMT process of IL-17 A-stimulated lung cells in vitro [84].

The Wnt/β-catenin signaling cascade is another pathway that drives EMT and contributes critically to lung cancer progression. This pathway is primarily governed by the equilibrium between β-catenin’s phosphorylation or dephosphorylation and its proteolytic degradation. In the cytoplasm, β-catenin associates with cadherin, undergoes phosphorylation by GSK-3β, and is ultimately degraded via the ubiquitin-proteasome mechanism [85]. Additionally, the IL-17B-IL-17RB signaling axis stimulates ERK phosphorylation, resulting in GSK3β inactivation, accumulation of β-catenin, and promotion of EMT in tumor cells [86].

Bone represents one of the most frequent sites for lung cancer metastasis, particularly in the case of lung adenocarcinoma, which exhibits the highest incidence of bone involvement (~ 40%) and is predominantly associated with osteolytic lesions that markedly compromise both survival and quality of life in patients. Osteoclasts are recognized as central mediators of this metastatic process. Evidence indicates that lung cancer cells secrete IL-17 A, which suppresses apoptosis in osteoclast precursors and activates mature osteoclasts. These activated osteoclasts then degrade the bone matrix and perpetuate a malignant cycle, thereby facilitating tumor colonization of bone tissue [87].

In summary, IL-17 plays a multidimensional and multi-level key regulatory role in the invasion and metastasis of lung cancer. Its pro-metastatic effects are primarily achieved through two core mechanisms: First, by activating key signaling pathways, it upregulates the expression of matrix metalloproteinases, disrupting the extracellular matrix (ECM) structure to clear physical barriers for tumor cell migration and invasion, Second, it drives epithelial-mesenchymal transition (EMT) by regulating key complexes and associated pathways, endowing tumor cells with enhanced motility and invasiveness. Notably, IL-17 not only acts on tumor cells themselves but also actively participates in constructing a pro-metastatic microenvironment—for example, by activating osteoclasts to promote bone metastasis in a vicious cycle. These findings underscore IL-17’s pivotal role as a molecular bridge linking chronic inflammation to tumor metastasis. However, most current research remains focused on deciphering individual pathways, with limited systematic understanding of how IL-17-driven signaling networks synergistically integrate spatially and temporally. Future studies should leverage spatial transcriptomics, in vivo imaging, and conditional knockout models to elucidate the dynamic roles of IL-17 in critical steps such as pre-metastatic niche formation, circulating tumor cell survival, and distant metastasis. Furthermore, targeting the IL-17 signaling axis—such as IL-17 A, IL-17RB, or its downstream effectors—may offer novel therapeutic strategies for inhibiting lung cancer metastasis, particularly refractory bone metastases. Thus, comprehensively deciphering the IL-17-mediated invasion and metastasis network not only deepens our understanding of lung cancer progression mechanisms but also opens new prospects for developing anti-metastatic interventions.

### IL-17’s role in angiogenesis and lymphangiogenesis in lung cancer

VEGF functions as a pivotal pro-angiogenic molecule, enhancing vascular permeability while promoting endothelial cell migration, proliferation, and new vessel formation [88, 89]. In the context of tumors, VEGF derived from malignant cells supports both tumor expansion and metastatic dissemination by increasing vascular density within the tumor microenvironment [90, 91]. According to the research, IL-17 facilitates angiogenesis and in vivo growth of murine NSCLC through mechanisms dependent on CXCR2, potentially mediated by elevated levels of angiogenic CXC chemokines, including CXCL1, CXCL5, CXCL6, and CXCL8 [92]. Elevated serum IL-17 concentrations in NSCLC patients have also been positively correlated with VEGF levels, indicating that IL-17-induced angiogenesis may play a role in disease progression [93]. Consistently, analyses of exhaled breath condensates from NSCLC patients revealed concomitant increases in IL-17 and VEGF, with a significant positive association between the two [94, 95].

On a mechanistic level, IL-17 has been shown to trigger STAT1 phosphorylation in lung adenocarcinoma cells, which leads to upregulation of IL-6 and IL-8, enhanced VEGF expression, and increased angiogenic activity [96, 97]. Similarly, activation of the STAT3/GIV pathway by IL-17 in NSCLC cells results in elevated VEGF production and augmented tumor-associated angiogenesis [98]. Another line of research indicates that IL-17 A suppresses glycolytic metabolism and glucose uptake in endothelial cells, while simultaneously enhancing fatty acid oxidation and mitochondrial energy output. This metabolic reprogramming, potentially mediated by AMPK phosphorylation and upregulation of ApoE and COX5A, provides the ATP and nucleotide precursors essential for angiogenesis [99].

Endostatin, a clinically utilized anti-angiogenic agent targeting VEGF, has been shown to significantly reduce IL-17 expression in tumor tissues when administered in a murine lung cancer model. This finding indicates that IL-17 and VEGF are closely associated with lung cancer, forming a mutually regulating positive feedback loop that jointly drives tumor progression. Therefore, when using Endostatin—an anti-angiogenic drug targeting VEGF—it not only directly inhibits the VEGF pathway but may also indirectly suppress IL-17 expression by disrupting this positive feedback loop. The significant reduction in IL-17 levels within tumor tissues observed in mouse lung cancer models treated with Endostatin provides compelling evidence for this bidirectional regulatory relationship. This highlights the synergistic role of IL-17 and VEGF in the pathological process of lung cancer, suggesting that jointly targeting these two molecules may represent a more effective therapeutic strategy [100].

In lung cancer, lymphatic spread constitutes an additional critical pathway for distant metastasis. Research has revealed that VEGF levels are closely associated with lymphatic metastasis in lung cancer, with VEGF-C serving as a key mediator in tumor-associated lymphatic vessel formation. More recent studies have revealed a strong association between IL-17 and VEGF-C expression and lymphatic vessel density (LVD) in non-small cell lung cancer. IL-17 enhances VEGF-C production in tumor cells, which then acts on lymphatic endothelial cells to drive lymphangiogenesis and facilitate metastatic progression [101]. Collectively, these findings underscore the potential of IL-17 as a therapeutic target for inhibiting lung cancer dissemination via both vascular and lymphatic routes.

Existing research collectively demonstrates that IL-17 not only participates in regulating the immune microenvironment of lung cancer as a pro-inflammatory cytokine, but also drives the remodeling of the tumor vasculature and lymphatic system through multidimensional mechanisms, serving as a pivotal link between chronic inflammation and tumor progression. Its pro-angiogenic function does not operate in isolation, but is embedded within a complex regulatory network comprising VEGF, CXC chemokines, and multiple signaling pathways. Notably, the positive feedback loop between IL-17 and VEGF reveals a profound interaction mechanism between inflammation and angiogenesis: inflammatory signals amplify the angiogenic response, while newly formed blood vessels in turn support inflammatory cell infiltration and survival, creating a self-reinforcing vicious cycle. The disruption of this dynamic equilibrium may serve as a critical driver for tumors transitioning from an inert state to an invasive phenotype. Furthermore, IL-17-mediated metabolic reprogramming of endothelial cells suggests its proangiogenic effects depend not only on classical growth factor signaling but also involve fine-tuned regulation of energy metabolism, offering new insights into the abnormal structure and function of tumor vasculature. At the lymphatic metastasis level, IL-17 directly promotes lymphangiogenesis by inducing VEGF-C expression, highlighting its strategic role in determining tumor metastasis pathways. Crucially, the bidirectional regulation of IL-17 expression by anti-angiogenic therapies (e.g., Endostatin) provides the first intervention-level validation of the plasticity within this inflammation-vascular axis, suggesting that targeting a single molecule may trigger cascading biological effects. Future research urgently requires constructing a more comprehensive IL-17 signaling map to clarify its heterogeneous functions across different cell types (e.g., tumor cells, stromal cells, endothelial cells) and explore its synergistic regulatory mechanisms with other immune checkpoints. Building upon this foundation, developing combined strategies that simultaneously block IL-17 and VEGF/VEGF-C pathways may overcome the current bottleneck of drug resistance in anti-angiogenic therapies, offering lung cancer patients treatment options with more durable efficacy.

IL-17 is not only a key factor driving chronic inflammation in the lungs, but also plays a crucial role in regulating the generation of blood vessels and lymphatic vessels through multi-dimensional regulation, becoming a core hub connecting inflammation and the progression of lung cancer. Its angiogenic-promoting effect is not an isolated event, instead, it activates signaling pathways such as STAT1/3, induces the expression of CXC chemokines and VEGF/VEGF-C, and collaborates with metabolic reprogramming to construct a complex tumor-promoting microenvironment network. Particularly crucial is the positive feedback loop between IL-17 and VEGF. Anti-angiogenic treatment can reverse the inhibition of IL-17, revealing the dynamic plasticity of the inflammatory-vascular axis.

### IL-17 and its role in the diagnosis and prognosis of lung cancer

Lung cancer remains the leading cause of cancer-related mortality globally, with a 5-year survival rate consistently falling below 15%, reflecting a poor prognosis. One reason for its high mortality rate is that by the time patients discover condition and receive a diagnosis, the disease has already progressed to an advanced stage. Currently, the commonly used methods for diagnosing lung cancer include X-ray, CT, PET-CT, and needle biopsy. Therefore, to significantly increase the proportion of health screenings among the general population and provide patients with more accurate and detailed diagnoses to guide clinical treatment, it is crucial to develop an efficient, precise, and minimally invasive method for early diagnosis and prognosis assessment.

High IL-17 expression has been shown to be independently linked with poorer overall survival (OS) and shorter disease-free survival (DFS) in patients with lung cancer. Stratifying further based on factors such as lung cancer histology (including non-small cell lung cancer and small cell lung cancer), tumor staging (I-III, I-IV, and IV), sample types (serum, tumor tissue, and pleural effusion), detection techniques (immunohistochemistry and ELISA), and HR estimation methods (reported and estimated) yielded statistically significant results [102]. These findings suggest that higher IL-17 levels are associated with worse clinical outcomes in lung cancer.

Carcinoembryonic antigen (CEA), a commonly used biomarker for lung cancer, is found to be elevated in the serum of NSCLC patients. Furthermore, circulating IL-17 A levels are also increased in these patients compared to healthy controls. The diagnostic performance of circulating IL-17 A and CEA was evaluated using ROC-AUC, and IL-17 A demonstrated a significantly superior AUC compared to CEA. Additionally, IL-17 A showed better diagnostic accuracy for early-stage NSCLC (stages I and II), outperforming CEA, which confirms its potential for accurate and reliable early diagnosis of NSCLC.

However, it should be noted that the elevation of IL-17 is not unique to lung cancer. It is also significantly increased in other inflammatory lung diseases such as severe chronic obstructive pulmonary disease (COPD) [103], active pneumonia or idiopathic pulmonary fibrosis (IPF) [104]. The lack of disease specificity limits the utility of IL-17 as an independent diagnostic marker. Therefore, a composite biomarker combination combining IL-17 with CEA and PD-L1 may be necessary to achieve sensitivity and specificity for clinically meaningful early detection or differential diagnosis.

As of 2023, LUAD has been identified as the most prevalent subtype of NSCLC. In LUAD, IL-17 and its downstream targets, including p-STAT3, CEA, and CA125, are expressed together. The combination of IL-1 and p-STAT3 has been shown to enhance the prognostic significance of CEA and CA125 for LUAD patients [105].

Surgical resection remains the standard treatment for early-stage NSCLC, however, the postoperative lymph node metastasis rate is around 11%, with a 5-year survival rate of approximately 66%. Factors such as surgical stress, sympathetic nervous system activation, ischemia-reperfusion injury, hypercoagulability, inflammation, and immune suppression can contribute to tumor metastasis. Lidocaine, a local anesthetic, has been shown to alleviate postoperative acute pain and inflammation. While some studies indicate that intravenous lidocaine infusion is associated with improved long-term survival following pancreatic cancer surgery, its role in NSCLC remains uncertain. A study involving early-stage NSCLC patients who received intravenous lidocaine compared to saline revealed that the lidocaine group had lower serum levels of IL-17 and cortisol. However, due to the short follow-up and the inclusion of early-stage patients, no significant differences were observed in recurrence or mortality rates [106]. γδT cells, which are essential for innate immunity, are notably abundant in barrier tissues and constitute about 8–20% of resident pulmonary lymphocytes. Given their developmental and functional plasticity, γδT cells exhibit varying roles across different tumor types. Studies have shown that γδT17 cells, which produce IL-17, are particularly enriched in LUAD and LUSC patients, especially among the elderly, where there is a decrease in CD4 + T cells, CD8 + T cells, and CD56 + cells. The high accumulation of γδT17 cells is linked to improved 3-year and 5-year overall survival (OS) in LUAD patients aged ≥ 60 years, and better 5-year OS in LUSC patients of the same age. Additionally, studies have shown that the elevated expression of aging markers such as LTBR, HES1, and RORC in the tumor microenvironment (TME) supports γδT17 cell accumulation. This suggests that intrinsic alterations in aging lung tissue may foster the enrichment of γδT17 cells, which could play an antitumor role in elderly individuals [107].

Immunotherapy has become an essential treatment for lung cancer, though no clear biomarkers are yet available to predict its safety and effectiveness. A prospective study involving 221 lung cancer patients receiving first-line or second-line PD-L1-based immunotherapy found that lower serum levels of IL-10 and IL-17 were linked to clinical benefits following the second treatment cycle [108].

Chronic pulmonary diseases in lung cancer patients significantly contribute to diminished anti-tumor efficacy and an increased risk of mortality. These conditions are mainly characterized by persistent airway inflammation, which is driven by various inflammatory mediators. As the understanding of how these mediators influence tumor progression deepens, there is increasing interest in identifying potential biomarkers for prognosis in lung cancer, especially in patients with chronic pulmonary conditions.

For instance, idiopathic pulmonary fibrosis (IPF) is well-established as a major risk factor for the progression of lung cancer. The incidence of lung cancer in IPF patients ranges between 10% and 40%, with these individuals generally facing poor prognoses. Studies have indicated that serum IL-17 levels in lung cancer patients with IPF are significantly higher than those found in patients with lung cancer alone. This provides further evidence supporting IL-17 as a potential biomarker for the early detection of lung cancer in IPF patients [109].

Chronic obstructive pulmonary disease (COPD) is another prevalent condition that impairs lung function by limiting airflow, leading to breathing difficulties. COPD shares common risk factors with lung cancer, such as smoking and exposure to occupational dust. Research has shown that IL-17 expression is significantly elevated in the alveolar and small airway walls of patients suffering from both COPD and lung cancer. This suggests a key role for IL-17 in promoting alveolar damage, airway inflammation, and tumor progression, although further studies are needed to elucidate the precise mechanisms.

Obstructive sleep apnea (OSA) has been associated with an increased risk of lung cancer-related mortality, though the underlying molecular mechanisms are not fully understood. A study on Lewis lung cancer (LLC)-bearing mice exposed to chronic intermittent hypoxia (CIH) or normal oxygen revealed that CIH exposure led to the upregulation of 207 genes and downregulation of 181 genes, compared to the normal oxygen group. Among the upregulated genes were those associated with IL-17 and TGF-β signaling pathways, suggesting that IL-17 signaling plays a role in the hypoxic physiological and pathological processes in lung cancer and may be associated with poor prognosis [110].

The association between IL-17 and adverse oncologic outcomes extends beyond lung cancer, highlighting a broader pathogenic role of this cytokine in epithelial carcinogenesis. Notably, IL-17 A has been shown to promote tumor growth in breast cancer and correlates with poor clinical outcomes in colorectal, breast, and lung cancers, underscoring its conserved pro-tumorigenic function across multiple solid tumors [111]. This cross-cancer evidence reinforces the biological plausibility of targeting IL-17 in lung cancer and provides a compelling rationale for investigating its mechanistic contributions within the lung tumor microenvironment (TME).

Importantly, the effects of IL-17 are not static, but exhibit distinct temporal and spatial heterogeneous dynamics. In the early stages of disease, IL-17 may initially play an immune surveillance or tissue repair function, thereby delaying malignant transformation. However, as tumors develop, IL-17 signaling shifts to promote angiogenesis, immunosuppression, and epithelial-mesenchymal transition, thereby becoming a driver of tumor progression. This dynamic highlights that IL-17 blockers may be beneficial in advanced or metastatic disease, but may inadvertently compromise protective immunity if applied indiscriminately at an early stage.

The potential risks of targeting IL-17 signaling pathways as a treatment strategy for lung cancer must be carefully evaluated. Although targeting IL-17 has demonstrated success in various inflammatory and autoimmune diseases, its application in cancer treatment—particularly in tumor patients whose immune function may already be compromised—requires heightened vigilance. Targeting IL-17 blockade may lead to increased infection risk, compromised intestinal barrier function and dysbiosis, disrupted immune homeostasis, and unintended tumor-promoting effects.

### Targeting IL-17 in lung cancer therapy

Although IL-17-targeting drugs are not yet available for clinical use in lung cancer treatment, numerous preclinical studies have shown that targeting IL-17 can significantly inhibit the progression of lung cancer. IL-17 A-targeting agents, for example, have demonstrated encouraging efficacy in treating autoimmune diseases such as psoriasis. Considering the limited effectiveness of immune checkpoint inhibitors in lung cancer therapy, combining IL-17 blockers with immune checkpoint suppression (ICS) has gained considerable attention as a promising area of research.

Recent evidence supports the therapeutic synergy and safety potential of IL-17 blockade in the context of immunotherapy. A single-cell sequencing study in a tumor mouse model revealed that combining IL-17 blockade with checkpoint blockade constitutes an effective strategy, with IL-17 inhibition significantly suppressing tumor progression by reshaping the tumor immune microenvironment [112]. Notably, emerging clinical data indicate that anti-IL-17 therapy can alleviate immune-related adverse events, particularly colitis and myocarditis, in patients receiving immune checkpoint inhibitors, highlighting its role in improving the safety profile of immunotherapy [113].

The role of IL-17 in mediating resistance to various lung cancer therapies—ranging from chemotherapy and radiotherapy to targeted agents and immunotherapies—has emerged as a critical theme in recent research. While platinum-based chemotherapies remain a cornerstone of non-small cell lung cancer (NSCLC) treatment, their efficacy is frequently undermined by acquired resistance. Although direct evidence linking IL-17 to classical chemoresistance remains limited, related interleukins such as IL-25 have been shown to activate the NF-κB pathway in cisplatin-resistant adenocarcinoma cells, suggesting broader involvement of the IL family in treatment failure [114]. More compellingly, IL-17 has been mechanistically tied to resistance against molecularly targeted therapies.

KRAS-activating mutations occur in approximately 30% of lung adenocarcinoma (LUAD) patients, with MEK serving as a key downstream effector in the KRAS-driven MAPK signaling pathway. Preclinical studies demonstrate that combining MEK inhibitors (MEKi) with anti-PD-L1 therapy significantly suppresses tumor growth and metastasis. However, prolonged treatment often leads to adaptive resistance, characterized by increased infiltration of Th17 cells and elevated IL-17 secretion within the tumor microenvironment. Notably, co-administration of IL-17 neutralizing antibodies restores therapeutic sensitivity, supporting the potential of a triple-combination strategy (MEKi + anti-PD-L1 + anti–IL-17) to overcome this resistance [115].

In the realm of immunotherapy, high levels of IL-17 are associated with diminished responses to PD-1/PD-L1 blockade. Overexpression of IL-17 A or IL-17 C in murine lung cancer models promotes tumor growth through upregulation of pro-inflammatory cytokines (e.g., IL-6, G-CSF) and enhances PD-L1 expression on tumor cells via inhibition of autophagy through the ROS/Nrf2/p62 axis, thereby fostering an immunosuppressive environment [28, 116, 117]. These findings position IL-17 not only as a driver of immune evasion but also as a key modulator of resistance to immune checkpoint inhibitors.

Beyond therapeutic resistance, IL-17 plays a pivotal role in treatment-related toxicity. Radiation-induced lung injury (RILI), a major limitation of thoracic radiotherapy affecting up to 70% of patients, is closely linked to IL-17-mediated inflammation. Radiation upregulates aquaporin 4 (AQP4) and HMGB1, triggering inflammatory cell infiltration and IL-17 production, which exacerbates tissue damage. IL-17–deficient mice exhibit reduced RILI severity, underscoring its pathogenic role [118]. Furthermore, when radiotherapy is combined with anti-PD-1 therapy, IL-17 A expression in Th17 cells is further amplified, worsening lung injury—a phenomenon attributed to the loss of PD-1–mediated restraint on Th17 hyperactivation [119]. Similarly, in checkpoint inhibitor pneumonitis (CIP), a serious immune-related adverse event, IL-17 A levels are elevated in both serum and bronchoalveolar lavage fluid of NSCLC patients and decline upon clinical resolution, implicating IL-17 A in the onset and progression of CIP [120]. Thus, IL-17 emerges as a dual player: contributing to both treatment resistance and therapy-induced toxicity.

Endotux, a recombinant human vascular endothelial inhibitor, exerts anti-angiogenic effects by blocking the VEGF signaling pathway. Recent studies have indicated that Endotux may also serve as a potential IL-17 inhibitor. When used in combination with anti-PD-1, it has been shown to notably suppress tumor growth in the LLC mouse model. The observed synergistic effect may be due to various factors, including a reduction in the levels of the pro-inflammatory cytokine IL-17 and the immunosuppressive factor TGF-β1, a decrease in MDSC enrichment, a reduction in CD8 + T cell suppression, and an enhancement in autophagy and apoptosis of tumor cells mediated through the PI3K/AKT/mTOR pathway [121].

Dimethyl seleno bis-propanoic acid (DSePA), a novel selenium-cysteine derivative, has shown potential in a mouse lung cancer model. It has been demonstrated to preserve glutathione peroxidase activity while inhibiting the NF-kB/IL-17/G-CSF/neutrophil axis, thereby preventing radiation-induced lung injury (RILI) caused by radiotherapy (RT). Furthermore, DSePA protects healthy lung tissue without affecting the radiation sensitivity of tumors, positioning it as a promising candidate for RILI prevention [122].

Beyond pharmacological inhibition, modulation of IL-17 signaling through epithelial-immune crosstalk also holds therapeutic relevance. Specific knockdown of neuropilin-1 (NRP1) in alveolar epithelial type II cells has been shown to inhibit TGF-β signaling, reduce Treg levels, and decrease expression of IL-17 A and IFN-γ, shifting Th cell polarization toward a Th2 phenotype and thereby mitigating RILI [123]. This effect is further supported by in vivo evidence demonstrating a positive correlation between IL-17 A levels and the severity of RILI. Mechanistically, IL-17 A exacerbates lung injury by suppressing autophagy via the PP2A B56α-mTOR pathway, underscoring its pathogenic role in treatment-related toxicity [124].

Chronic inflammation is a significant factor in promoting lung cancer development, with inflammatory cells such as Th17 cells and macrophages playing a crucial role in driving tumor progression. IL-10, a versatile immunoregulatory cytokine, is vital for sustaining immune homeostasis within mucosal tissues. It facilitates the conversion of immature blood monocytes into tolerogenic macrophages, directly suppressing Th17 cell activity. In the LSL-K-rasG12D mouse model of lung cancer, intratracheal administration of a novel IL-10 formulation disrupts the macrophage-Th17 cell axis, effectively inhibiting tumor growth driven by IL-17^+^CD4^+^T cells. These findings offer new evidence supporting the potential role of IL-10 inhalation in preventing lung cancer in high-risk populations [125].

Symbiotic bacteria are found in various regions of the body, including the respiratory and gastrointestinal tracts. They are critical for preserving immune balance in mucosal tissues, such as those in the lungs and intestines. A deficiency in these bacteria can lead to developmental defects in the immune system, thereby increasing the likelihood of tumor formation. Extensive research has been conducted on the relationship between intestinal bacteria and colorectal cancer. Fecal microbiota transplantation, when combined with targeted therapy and immunotherapy, has demonstrated promising therapeutic outcomes in patients with colorectal cancer [126]. In contrast, research on the connection between lung bacteria and lung cancer is still in its early stages due to the complex diversity of bacterial species present in the lungs. Studies involving antibiotic-treated (Abt) mouse models have shown that Abt mice are more prone to developing LLC and tend to have a worse prognosis. Additionally, in Abt mice, a suppression of the γδT17 cell response was observed, and the restoration of the impaired anti-tumor response was achieved by adding normal γδT17 cells or supplementing with IL-17. This study has introduced a “new perspective,” suggesting that the IL-17 immune response, mediated by symbiotic bacteria, plays an essential role in maintaining immune homeostasis and inhibiting tumors, thus providing new evidence for the potential of lung bacteria as anti-cancer agents [127].

The clinical translation of IL-17-targeting strategies will require careful patient stratification, and subgroups of potential benefit may include patients with cancer, those with immune checkpoint inhibitor-related toxicity, or those with comorbid COPD/IPF and elevated IL-17 levels. Biomarkers included serum IL-17 A, tumor-infiltrating Th17/γδT17 cells, or IL-17 co-expression with VEGF/PD-L1. Moreover, the optimal timing of intervention depends on the context: blockade is often not appropriate in the earliest stages of disease, whereas blockade may be most beneficial in mitigating treatment-related toxicity or overcoming resistance in advanced disease.

In summary, IL-17 functions through different therapeutic modes: (1) In chemotherapy, IL-17 family members such as IL-25 may activate the NF-κB signaling pathway to enhance cisplatin resistance, (2) In targeted therapy, IL-17 A/IL-17RC signaling promotes the phosphorylation of EGFR and MET in EGFR-mutant NSCLC, and drives EGFR-TKI bypass resistance. (3) During radiotherapy, IL-17 may aggravate radiation-induced lung injury by maintaining inflammatory response and inhibiting autophagy through PP2A B56α-mTOR pathway, (4) In immunotherapy, IL-17 can cultivate an immunosuppressive niche by up-regulating PD-L1 and inhibiting CD8 + T cell function to weaken the checkpoint blockade response.

Overall, although IL-17-targeted drugs have yet to be clinically applied in lung cancer treatment, extensive preclinical research has conclusively demonstrated that targeting the IL-17 signaling pathway holds multi-level, multi-strategy intervention potential in lung cancer therapy. Its core lies in breaking the vicious cycle between the pro-inflammatory microenvironment and tumor progression. Current research has moved beyond the simplistic linear thinking of “inhibition equals benefit,” instead exploring more refined and synergistic therapeutic paradigms. A key insight is that IL-17 not only drives tumor immune evasion and metastasis but also serves as a critical mediator for resistance or toxicity to various treatments, such as MEK inhibitors and radiotherapy. Therefore, employing IL-17 blockade as a “synergistic and toxicity-reducing” combination strategy holds immense clinical translational value. These approaches collectively point toward a core principle: restoring or rebuilding the body’s immune homeostasis, rather than merely eliminating a single component. However, targeted IL-17 blockade may increase infection risks, compromise intestinal barrier function and cause dysbiosis, disrupt immune homeostasis, and trigger unintended pro-tumor effects—particularly elevating upper respiratory infection risks in lung cancer patients. Consequently, maintaining vigilance regarding IL-17’s double-edged biological properties is crucial for advancing precision and safety in this field. Future progress must be grounded in deep analysis of the IL-17 signaling network. Technologies like single-cell omics and spatial transcriptomics should be leveraged to precisely map its dynamic profiles across different patients and disease sites. Building on this foundation, companion diagnostic tools must be developed to enable precise decision-making regarding “when to intervene,” “who to intervene in,” and “how to combine therapies.” Ultimately, this field requires bold clinical exploration through mechanism-driven innovative trials to translate these promising preclinical discoveries into therapeutic solutions that tangibly improve survival and quality of life for lung cancer patients.

## Summary and conclusion

Interleukin-17 (IL-17) has increasingly garnered attention due to its role in the onset, progression, and treatment of lung cancer. As a cytokine that promotes inflammation, IL-17 significantly contributes to the advancement of lung cancer by regulating key processes, such as immune evasion by tumors, cell proliferation, invasion, metastasis, and angiogenesis. Research has demonstrated that IL-17 not only plays a critical role in modulating the immune microenvironment within lung cancer but also contributes to tumor resistance and unfavorable prognoses. Therefore, IL-17 has emerged as a key target in lung cancer diagnosis, prognosis assessment, and treatment strategies, demonstrating significant potential for clinical application.

However, the function of IL-17 in the tumor microenvironment exhibits significant duality, with its pro-tumor and anti-tumor effects highly dependent on cell origin, tissue microenvironment, and host immune status. This complexity has led to significant controversy regarding its use as a therapeutic target. Of particular note, targeting IL-17 may disrupt mucosal immune homeostasis and increase infection risk, a concern that is especially pronounced in lung cancer patients with concomitant chronic pulmonary disease or those receiving combination immunotherapy. Indeed, clinical experience from psoriasis and spondyloarthropathy trials with IL-17 A inhibitor has revealed a reproducible increase in the incidence of mild-to-moderate upper respiratory tract infections, including nasopharyngitis and bronchitis, and exacerbations of pre-existing inflammatory bowel disease. While serious opportunistic infections remain uncommon in otherwise immunocompetent populations, these signals are highly relevant for lung cancer patients, who are often elderly, immunocompromised due to disease or therapy, and frequently harbor comorbid COPD, pulmonary fibrosis, or subclinical lung infections. In such individuals, IL-17 blockade could impair neutrophil recruitment and antimicrobial peptide production at mucosal barriers, potentially converting indolent colonization into active pneumonia or worsening radiation- or immunotherapy-induced pneumonitis. Therefore, careful patient selection is paramount: baseline screening for active or latent respiratory infections, exclusion of uncontrolled interstitial lung disease, and vigilant monitoring during treatment are essential mitigation strategies. Moreover, transient or localized inhibition—rather than systemic ablation—of IL-17 signaling may offer a safer therapeutic window in this vulnerable population. Furthermore, the clinical strategy for combining IL-17 with other immune checkpoint inhibitors remains unclear. Critical questions regarding “which agents to combine, how to combine them, and when to combine them” urgently need resolution to balance therapeutic efficacy with immune-related adverse events. To shed light on the controversy, aligned comparative studies, such as conditional knockout models, should be conducted to investigate the spatiotemporal dynamics of IL-17 from different cell sources. Guided by healthy patient stratification and dynamic biomarker assessment, il-17 targeting strategies should be integrated into the precision medicine framework to maximize efficacy while minimizing risk. Future research should integrate cutting-edge technologies such as single-cell genomics, spatial transcriptomics, and bioinformatics to deeply decipher the spatiotemporal dynamics and cell-specific functions of IL-17 signaling, thereby advancing the development of precision intervention strategies. Furthermore, continued investigation into IL-17 and its associated signaling pathways will provide new insights and open avenues for improved strategies in the diagnosis, prevention, and treatment of lung cancer.

## Data Availability

No datasets were generated or analysed during the current study.
